# The experiences of balloon-expandable stent in symptomatic stenosis of middle cerebral artery

**DOI:** 10.1186/s40064-016-3078-4

**Published:** 2016-08-24

**Authors:** Lingtao Tang, Pengfei Hu, Yalin Liu, Kunxi Zhang, Yun Wang, Dong Qi, Zhongshuang Xia, Shanshan Qi, Suxia Zhang, Hongmei Zhang, Weiyue Li, Bogang Zhang, Xingdan Yang, Yingyi Li

**Affiliations:** The Cerebrovascular Disease Center, The Third Hospital of Xingtai, No. 108, Steel Road (North), Qiaoxi District, Xingtai City, 054000 Hebei Province China

**Keywords:** Middle cerebral artery, Stenosis, Balloon-expandable stent

## Abstract

**Background:**

Stent placement for middle cerebral artery (MCA) stenosis remains a technical and clinical challenge. Our purpose was to assess the safety and feasibility of balloon-expandable stent (BES) for patients with symptomatic M1 stenosis of MCA, and to introduce our experience during the procedure.

**Methods:**

In the study, we analyzed retrospectively 37 patients with M1 stenosis of the MCA ranged from 70 to 90 % in diameter reduction and refractory to medical therapy between January 2012 and January 2015. All the patients underwent angioplasty and stenting with BES, and followed up continuously.

**Results:**

Thirty-five out of 37 patients were successfully followed up and available until now. The technical successful rate was 100 % for all the lesions. The complication rate was 0 during the procedure. Stroke occurred to one patient at 4th day after the procedure. There were two patients experiencing slight stroke after 8 months. Two patients were found re-stenosis >50 % without any symptom. The stroke rate of 12 months was 8.57 % (3/35).

**Conclusions:**

Angioplasty associated with BES appears to be safe and feasible for the patients with symptomatic M1 stenosis of MCA. Our experiences about the BES may be valuable for decreasing the complication. However, further study is needed.

## Background

In Asia, Europe and North America, stroke is the third cause of death and disability (Thom et al. 2006; Donnan et al. 2008). Compared with the whites, primary atherosclerotic at MCA may play a significant role in Asians (Caplan et al. 1986; Li and Wong 2003). In Chinese population, intracranial atherosclerosis is more common (Suri and Johnston 2009). Symptomatic MCA stenosis is a common cause of ischemic stroke. Therefore, much attention has been paid in this research area in the past years in China.

Nowadays, the main treatments for the intracranial stenosis include medical therapy, percutaneous transluminal angioplasty (PTA) or percutaneous transluminal angioplasty and stenting (PTAS), and surgical intervention. But the optimal treatment remains controversial. There was a high rate of the stroke in the patients with MCA stenosis, even with the optimal medicine treatment (Chimowitz et al. 1995). So the therapeutic effect of expectant treatment with antiplatelet and anticoagulation is limited (Chimowitz et al. 2005). PTAS have been considered as an alternative treatment (Jiang et al. 2004, 2007; Kim et al. 2005; Suh et al. 2008). Stent placement was certified to be safe and efficient (Lee et al. 2005; Mohammadian et al. 2012; Zhang et al. 2012). Nowadays, there are two types stents used in intracranial atherosclerotic stenosis, one is self-expandable stent (SES), and the other is balloon-expandable stent (BES). Because of the flexibility of the SES, its’ usage was more widespread. But the SAMMPRIS trial (Chimowitz et al. 2011) and the VISSIT trial (Zaidat et al. 2015) revealed the negative results. Recently, the safety and efficacy were certified again (Miao et al. 2015).

The purpose of this study was to introduce the experience of BES for the patients with symptomatic MCA stenosis.

## Methods

Between January 2012 and January 2015, we retrospectively analyzed the medical records of 37 patients underwent PTAS using the BES (APOLLO, Micro-port Neuro Tech, China) for the atherosclerotic stenosis of MCA. The study was approved by the institutional review board, and the way of endovascular treatment was informed written consent by all the patients.

APOLLO is one type of balloon-expandable stent used to treat intracranial arterial stenosis, which is produced by MicroPort Scientific Corporation of China. The advantage of the stent is its supporting force and flexibility. The conveyor is the rapid exchange balloon catheter. It has excellent pushing and crossing ability.

The inclusion criteria of the study were as follows: recurrent transient ischemic attack (TIA) or stroke despite optimal medical therapy related to the symptomatic atherosclerotic stenosis in the M1 segment of MCA; the digital subtraction angiography (DSA) showing the stenosis ≥70 %; the type of lesion was Mori A or B (Mori et al. 1998); The age of the patients was between 30 and 75 years.

The exclusion criteria were as follows: non-atherosclerotic arterial stenosis; the patients with tumors, aneurysms, heart failure, hepatosis, kidney failure, ulcer, hematological system diseases, vasculitis, hyperthyroidism; allergic reaction for aspirin, clopidogrel and iodine; the lesion angulated <135 degree; patients with the severe neurological impairment could not take care of themselves in their life.

All patients were pre-medicated with doses of aspirin (100 mg/d) plus clopidogrel (75 mg/d) at least 5 days before the endovascular therapy.

The therapeutic procedures were performed during the second angiography. All endovascular procedures were performed under local anesthesia by a neuroradiologist. The right femoral artery was selected the percutaneous access. And an 8F sheath (APOLLO, Micro-port Neuro Tech, China) was inserted after the femoral puncture. Before the therapeutic procedure, the patients received systemic heparinization by a bolus injection of heparin 75 IU/kg. Then a 6F guiding catheter (APOLLO, Micro-port Neuro Tech, China) was inserted into the distal C2 of internal cerebral artery. Then a micro wire went straight through the guiding catheter to fix to the M2 of the MCA. If the access was tortuous and the stent could not get to the right position, the second micro wire was used to increase the support force. Under the road-map image, the stent passed through the stenosis carefully along the micro wire (If using the second micro wire, it should be pulled out at the time). Then use the “step-by-step” multi-step pressure technique to deploy the stent. The stent was delivered from the wire by dilating the balloon by 3 atm for 3 s firstly, then the pressure was increased to 4 atm for 3 s, 5 atm for 3 s to reduce the residue stenosis gradually. The maximum pressure was no more than 6 atm to avoid rupture.

All patients took orally clopidogrel (75 mg/d) for 1 year. Aspirin was used continuously (100 mg/d). Low-molecular-weight heparin calcium injection was used by subcutaneous injection every 12 h for at least 3 days. All patients were followed up continuously. Data analysis was performed by using Sigmaplot 10.0 statistical software.

## Results

We studied 37 patients (21 men and 16 women) ranging in age from 32 to 72 years (mean age 55.1 years). All patients experienced TIA (n = 6) or stroke (n = 31). All the stenosis of the M1 segment of MCA was directly responsible for the symptoms.

**Fig. 1:**
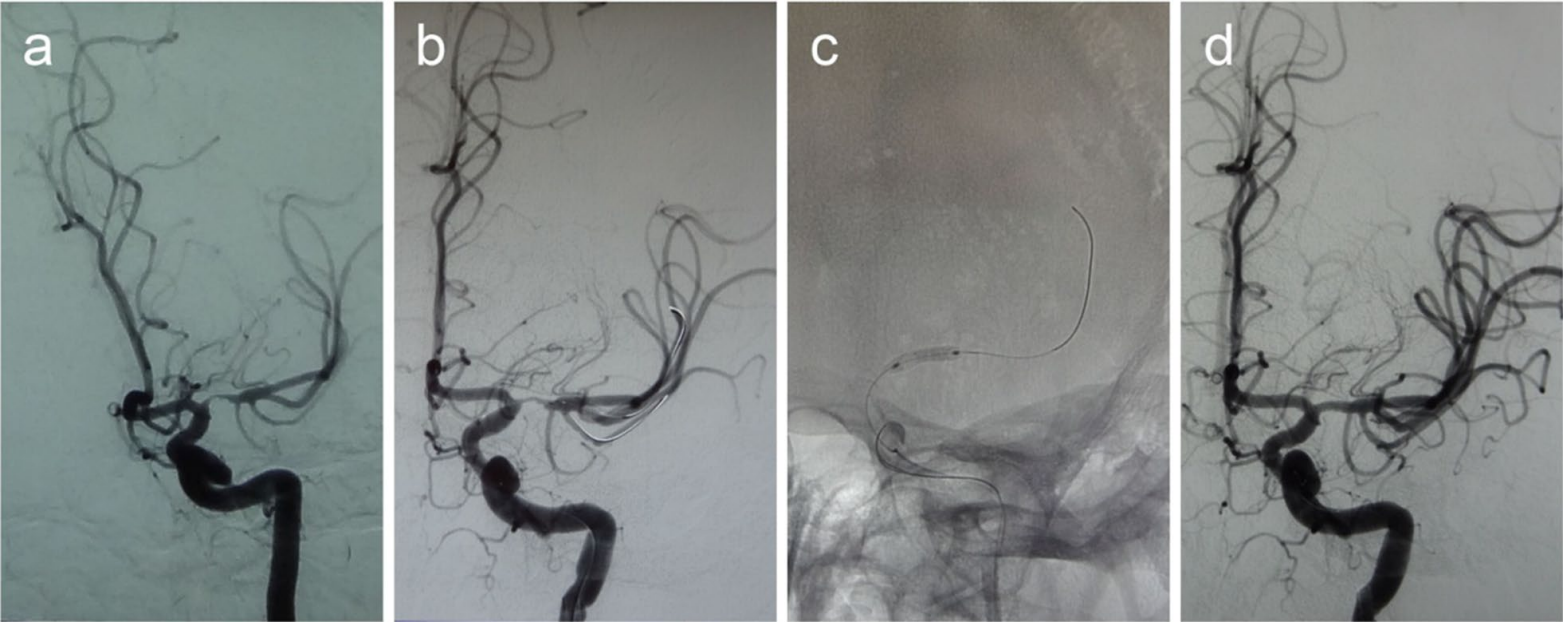
A 45-year-old man with a stroke. **a** DSA shows severe stenosis (about 90 %) in the M1 portion of the left MCA and tortuous pathway, **b** the double-micro-wire was fixed, **c** the stent is reaching to the right position and expanded gradually, **d** DSA shows recanalization of the diseased segment after the post-procedure

The technical successful rate was 100 %. For tortuous vessel, we used the double-micro-wire technique to increase the rate of the technique success (Fig. 1). Mean preprocedual stenosis was 80.27 % (range 70–90 %). Postprocedure the residual stenosis was 0–20 % (the mean of 7.30 ± 5.60 %). During the procedure, there was none complication, including thrombosis in stent, artery rupture, lenticulostriate artery occlusion.

**Fig. 2:**
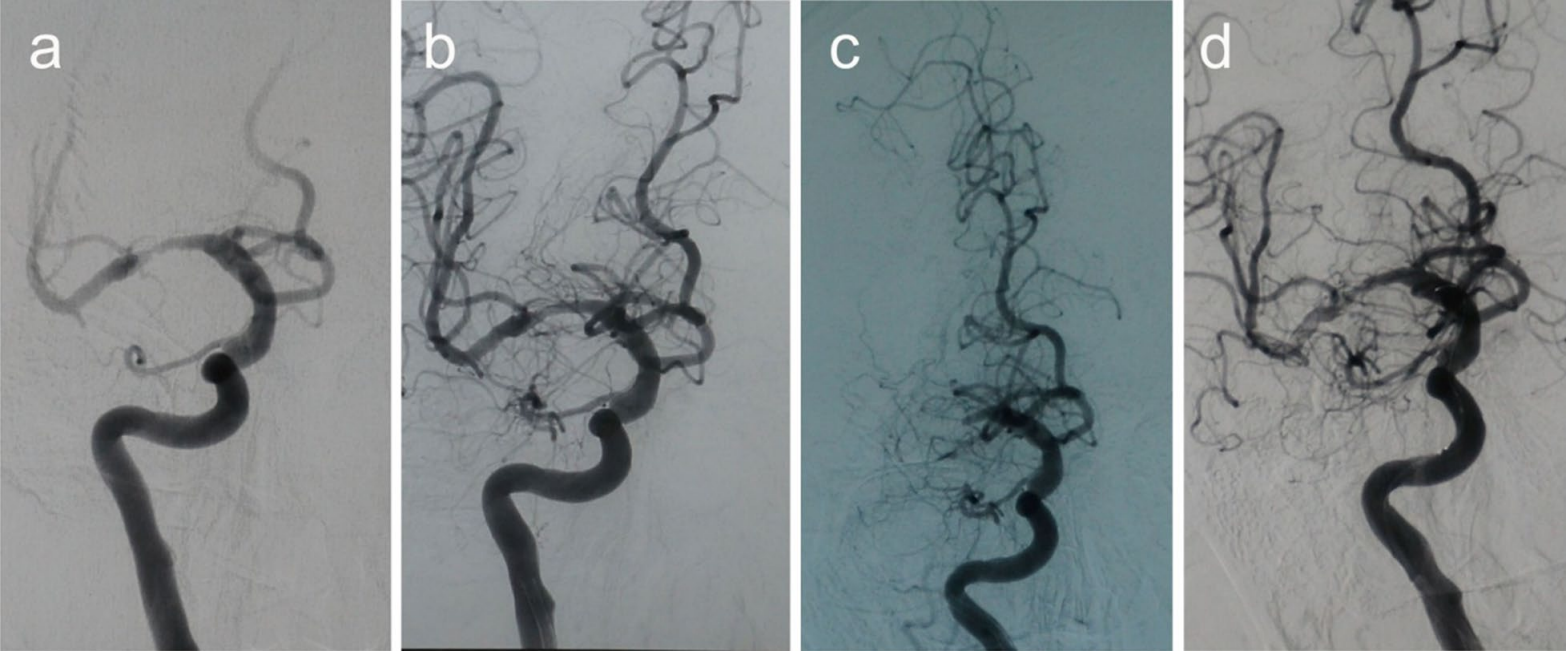
A 51-year-old man with a stroke. **a** DSA shows severe stenosis (about 80 %) in the M1 portion of the right MCA, **b** DSA shows recanalization of the diseased segment after the post-procedure, **c** DSA shows an acute thrombosis in the stent at 4-day after the procedure, **d** vascular recanalization using the micro-wire

**Fig. 3:**
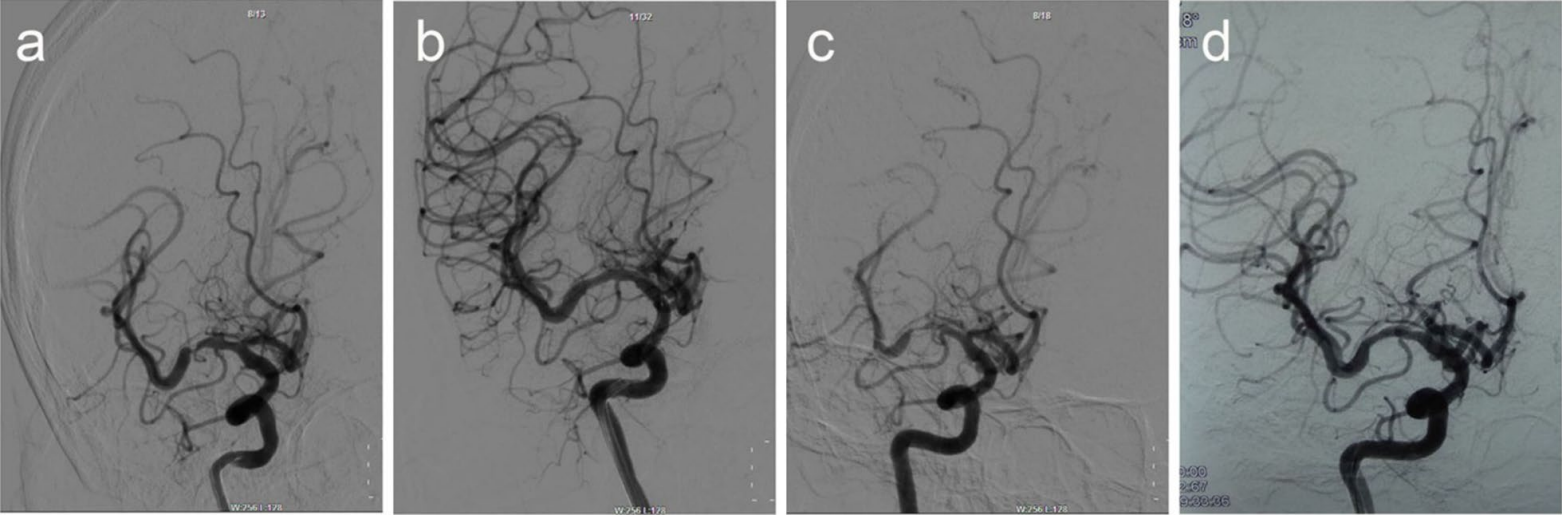
A 42-year-old woman with a stroke. **a** DSA shows severe stenosis (about 80 %) in the M1 portion of the right MCA, **b** DSA shows excellent recanalization of the diseased segment after the BES positioned at the lesion, **c** 8 months later, DSA shows that the degree of re-stenosis is more than 70 %. **d** the second BES is positioned to improve the lesion of the restenosis

**Fig. 4:**
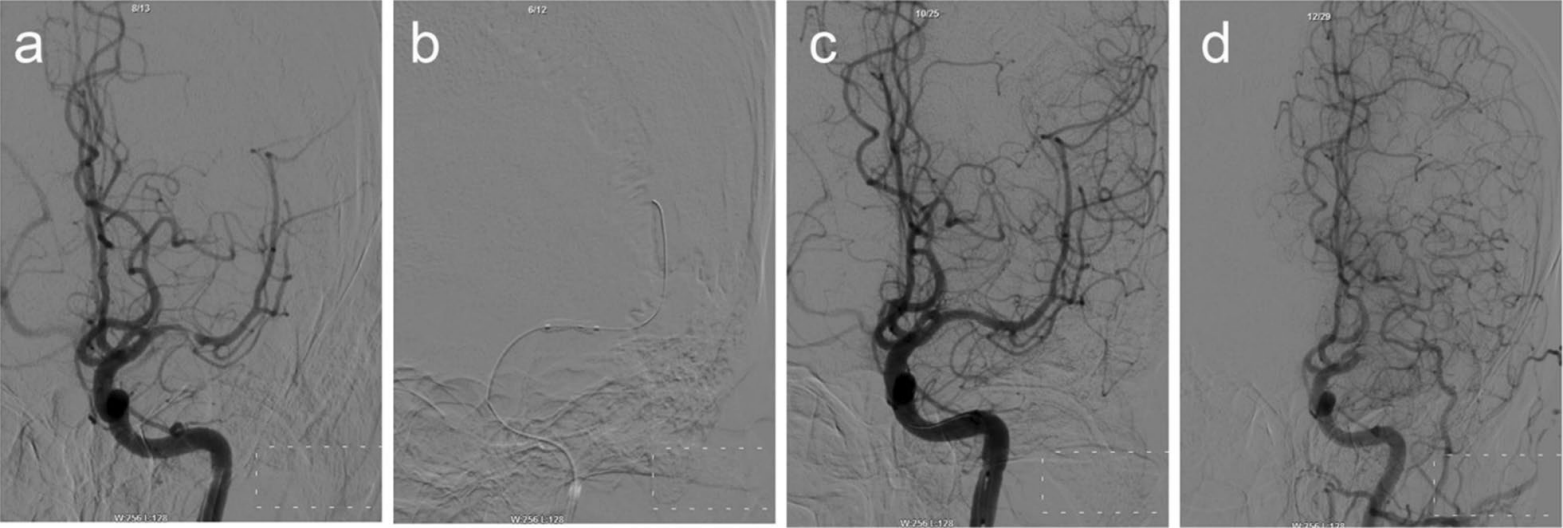
A 51-year-old man with a stroke. **a** DSA shows severe stenosis (about 90 %) in the M1 portion of the left MCA, **b** the BES is positioned at the lesion, and the micro wire is anchored in the distal branch of the MCA, then the balloon is expanded gradually, **c** DSA shows excellent recanalization of the diseased segment immediate post-procedural, **d** at the 8 months, the thrombus blocked the artery in the stent completely. But the vascular compensatory through pial artery was complete

**Fig. 5:**
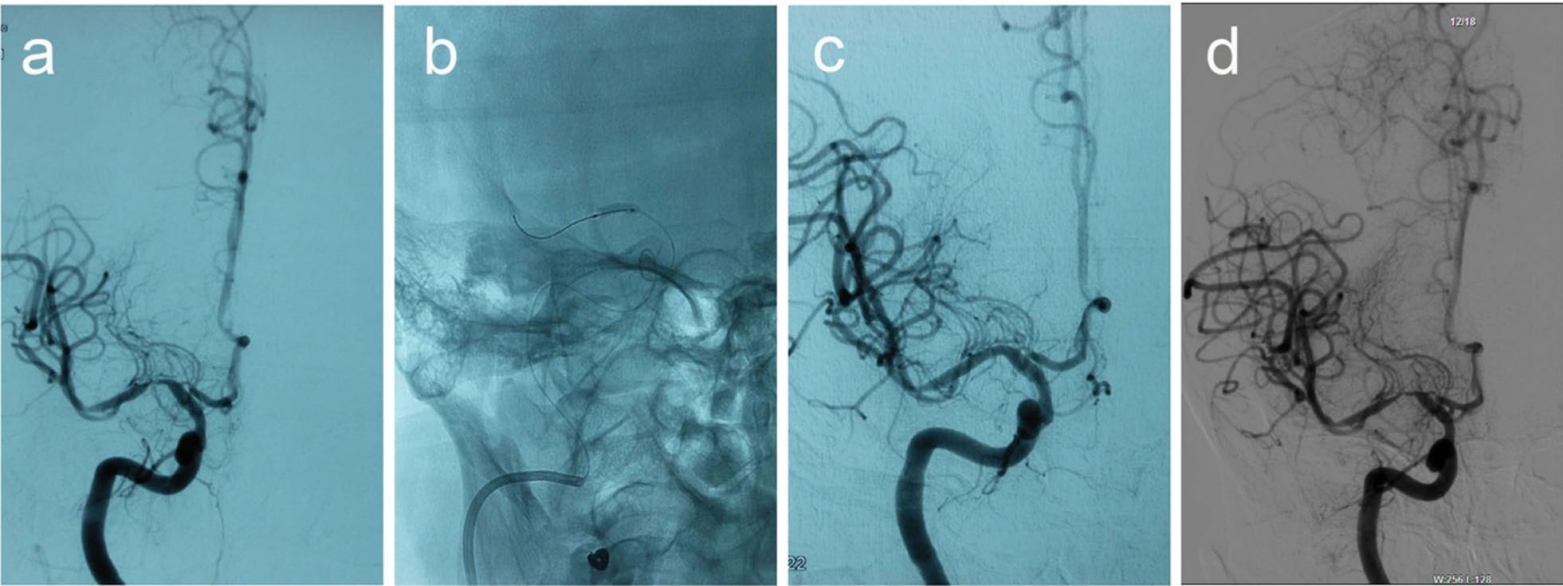
A 58-year-old woman with a stroke. **a** DSA shows severe stenosis (about 80 %) in the M1 portion of the right MCA, **b** the BES is positioned at the lesion, then the balloon is expanded gradually, **c** DSA shows excellent recanalization of the diseased segment immediate post-procedural, **d** 12 months later, DSA shows that the degree of re-stenosis is more than 50 %

**Fig. 6:**
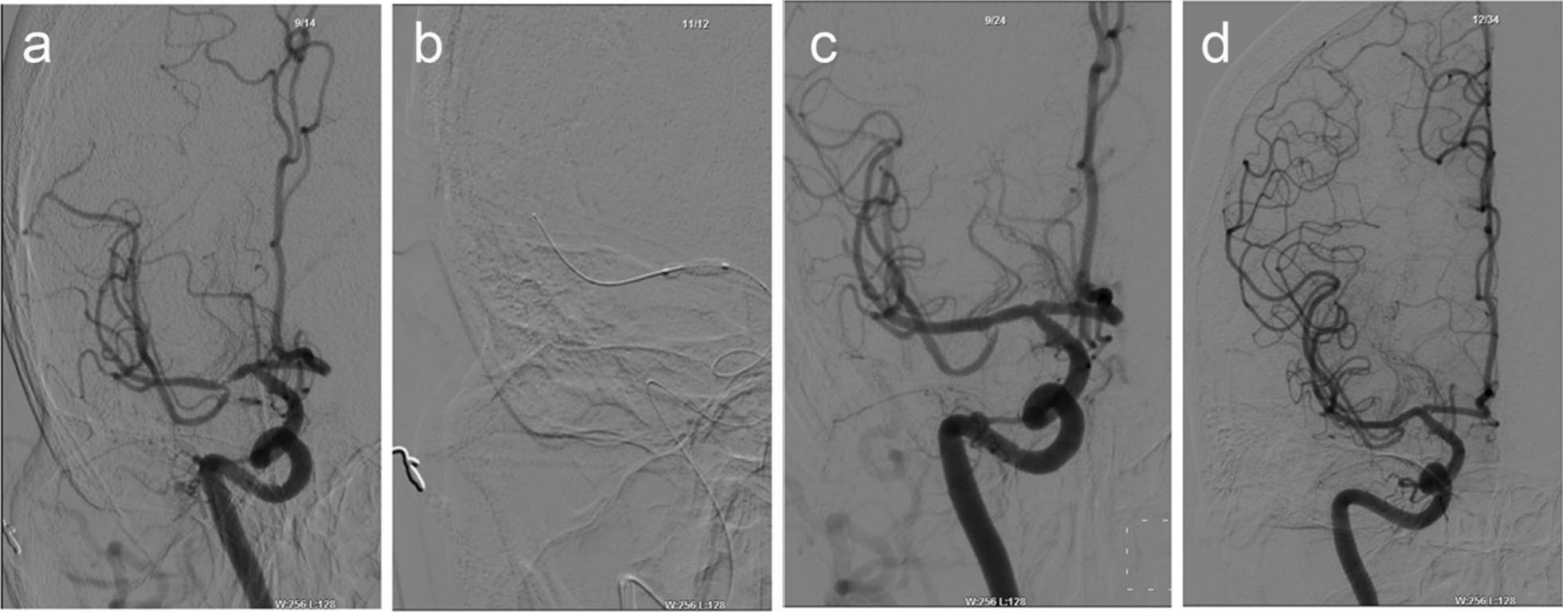
A 55-year-old woman with a stroke. **a** DSA shows severe stenosis (about 90 %) in the M1 portion of the right MCA, **b** The BES is positioned at the lesion, **c** DSA shows excellent recanalization of the diseased segment immediate post-procedural, **d** 36 months later, DSA shows a perfect result

Of the 37 patients who underwent PTAS, followed-up successfully were 35 people. Clinical follow-up was available up to now. 30-days stroke rate was 2.70 % (1/37). One patient occurred stroke at 4 days after the procedure. DSA certified acute thrombosis in the stent. We accessed the vascular successfully (Fig. 2). Micro-wire passed through the occlusion. Then we processed PTA. But there was more thrombus, then we used GPIIb/IIIa receptor antagonist (Tirofiban Hydrochlorid). There were two patients occurring slight stroke after 8 months. The one occurred re-stenosis >50 % (Fig. 3). The thrombus completely blocked in the stent in the other one (Fig. 4). But the neurological deficit symptom was disappeared completely by medical therapy. The rest did not show any stroke or TIA. The 12 months stroke rate was 8.57 % (3/35). However, two patients were found re-stenosis >50 % without any symptom (Fig. 5). Clinical follow-up for the patients with the longest time was 36 months. DSA showed a perfect result (Fig. 6).

## Discussion

The controversy about the treatment of the endovascular stenting for patients with symptomatic intracranial arterial stenosis is going on. Complication is one of the reasons. We used BES for the patients with MCA stenosis, and obtained satisfactory results. Using the multi-step technique could decrease the complication.

Gomez used the stent to treat the stenosis of MCA (Gomez et al. 2000). Soon after, the coronary BES was used to treat the MCA stenosis (Shin et al. 2003; Kim et al. 2004). Also, it was certified to be effective and feasible (The SSYLVIA Study Investigators 2004; Jiang et al. 2004, 2007; Fiorella et al. 2007). Especially the appearance of the Winspan stent (Henkes et al. 2005), the treatments of the symptomatic intracranial arterial stenosis are showing a new situation. However, the SAMMPRIS trial certified aggressive medical management was superior to PTAS (Chimowitz et al. 2011). The VISSIT trial did not support the use of BES (Zaidat et al. 2015). Nevertheless, Miao revealed the safety and efficacy of endovascular stenting (Miao et al. 2015). The reporters found that there was no significant difference between the SES and the BES about the follow-up clinical outcomes (Yue et al. 2011b). But the expense and the complicated procedure of the SES restricted the use of the Winspan stent (Zaidat et al. 2008), especially in developing countries. For this reason, we use the BES to treat the MCA stenosis.

The most severe complication was intracranial hemorrhage (Brus-Ramer et al. 2010). The rate was 7.5 % (Jiang et al. 2004). In addition, the perforating artery occlusion may cause severe cerebral infarction. Jiang found that the rate of perforating vessel occlusion near the site of stenosis of was 3.0 % (Jiang et al. 2006). In particular, perioperative complication rate was 10–20 % in the patients with BES (Chow et al. 2005). We used the APOLLO stent to treat the MCA stenosis. The successful rate of stent-angioplasty was 100 % in this study. No infarction or hemorrhage was observed during the procedure. During the procedure, we used the way of step-by-step to deploy the stent. The results indicated that the way was safe and effective. Firstly, it reduced the rate of the rupture of artery. During the first step, the stent gave the weak muscle fibers of the artery a buffer chance. Secondly, it made the atherosclerotic plaque “find” the position of them. So it avoided blocking the lenticulostriate artery. This was a hypothesis. The specific principle needs future study. Certainly, if there are obvious and more the lenticulostriate arteries nearby the lesion, it should be cautious to choose BES. The procedural complication is associated with the lesion with calcification or without. We did not confirm the lesion calcification or without, because there is no High Resolution Magnetic Resonance Imaging.

Vessel tortuosity may increase the technical difficulty thus raising the risks. Some investigators reported that the stent could not arrive at the right position due to the tortuous artery pathway (Kim et al. 2004). This was one of the major reasons of failure. In this condition, we used double-micro-wire to increase the support power. This way not only reduced the single micro wire inactivity bumped the tunica intimae, but also shortened the whole time of the procedure. This is another cause of the high success rate. For the longer lesion (Mori C) and excessively tortuous pathway, BES is not a better choice. After all, the BES is less flexible than SES.

Because the residual stenosis rate was one of the major factors affecting the restenosis (Yue et al. 2011a), so less residual stenosis is more beneficial. In the SES, the residual stenosis rate was over 10 % (Kim et al. 2012; Zhang et al. 2012) because of the balloon dilatation angioplasty without simultaneous stenting. The problem was resolved by the BES. BES may have a lower degree of residual stenosis than SES (Miao et al. 2015). We obtained an outstanding angiographic result with BES. In some cases, the residual stenosis was 0.

The SSYLVIA study reported the restenosis rate of ≥50 % at 6 months was 32.4 % (The SSYLVIA Study Investigators 2004). However, the investigators reported that there was no restenosis in the cases with drug-eluting stent (Lee et al. 2013). The restenosis is associated with the angle of the lesion. When the lesion was tortuous, the type of Mori C, the rate of stroke was 87 %, the rate of restenosis was 100 % (Mori et al. 1998). In this study, we had chosen the lesion angled >135 degree to decrease the rate of the restenosis. Two patients showed stroke at 8-month. One had restenosis over 50 %. The reason was that she did not take orally aspirin and clopidogrel regularly. We used another stent to treat the restenosis. Up to now, she showed none ischemic symptom. In the other case, the thrombus blocked the artery in the stent completely. There was not any other reason except for unhealthy lifestyle. But the vascular compensatory through pial artery was more complete, so the patient showed slight neurological impairment. Through drug treatment, he had none sequela. The stent provided collateral circulation buffering time. So even under the circumstances of the thrombosis in the stent, the collateral circulation could ensure adequate blood supply to the corresponding functional areas.

## Conclusions

In this study, BES appeared to be safe, effective and feasible for MCA stenosis. The whole procedure was simple. It may be suitable for Chinese and the patients of other developing countries. This is our experience of the BES. However, the number of the patients included was relatively small. Further study should be prompted on a large scale, multi-center research.

## Abbreviations

MCA: middle cerebral artery
BES: balloon-expandable stent
SES: self-expandable stent
PTA: percutaneous transluminal angioplasty
PTAS: percutaneous transluminal angioplasty and stenting
DSA: digital subtraction angiography
TIA: transient ischemic attack
